# The somatic *POLE* P286R mutation defines a unique subclass of colorectal cancer featuring hypermutation, representing a potential genomic biomarker for immunotherapy

**DOI:** 10.18632/oncotarget.11862

**Published:** 2016-09-06

**Authors:** Sung-Min Ahn, Adnan Ahmad Ansari, Jihun Kim, Deokhoon Kim, Sung-Min Chun, Jiyun Kim, Tae Won Kim, Inja Park, Chang-Sik Yu, Se Jin Jang

**Affiliations:** ^1^ Division of Hematology-Oncology, Department of Internal Medicine, Gachon University Gil Hospital, Incheon, South Korea; ^2^ Gachon Institute of Genome Medicine and Science, Gachon University Gil Hospital, Incheon, South Korea; ^3^ Department of Biomedical Engineering, University of Ulsan College of Medicine, Seoul, South Korea; ^4^ Asan Center for Cancer Genome Discovery, Asan Institute for Life Sciences, Asan Medical Center, Seoul, South Korea; ^5^ Department of Pathology, University of Ulsan College of Medicine, Asan Medical Center, Seoul, South Korea; ^6^ Department of Oncology, University of Ulsan College of Medicine, Asan Medical Center, Seoul, South Korea; ^7^ Department of Surgery, University of Ulsan College of Medicine, Asan Medical Center, Seoul, South Korea

**Keywords:** early-onset colorectal cancer, POLE, hypermutation, immunotherapy, PD-1 blockade

## Abstract

Early-onset colorectal cancers (EOCRCs) may have biological or genomic features distinct from late-onset CRCs (LOCRCs). Previous studies have mostly focused on the germline predisposition conditions of EOCRCs, but we hypothesized that EOCRCs may have distinct somatic aberrations that accelerate cancer development. To identify the somatic aberrations that accelerate cancer development at an early age, we conducted whole exome sequencing for 28 polyposis-unrelated, microsatellite stable (MSS) EOCRCs with no known germline predisposition conditions. Surprisingly, we found two distinct groups in the context of mutational burden: 6 hypermutated cases with 2325 to 10973 mutations and 22 nonhypermutated cases with 47 to 154 mutations. Further analysis revealed that four of the six hypermutated cases had the same *POLE* P286R mutation. We validated this finding in 83 MSS EOCRCs and 27 MSS LOCRCs, which revealed that 7.2% of EOCRCs (6/83) had the *POLE* P286R mutation, which was not found in LOCRCs. Clinicopathologically, EOCRCs with *POLE* mutations occurred far more frequently in the right colon than in the left colon, affecting men more frequently than women. In summary, we have identified a unique subclass of colon cancer characterized by a hypermutation associated with the *POLE* mutation. The acquisition of the *POLE* mutation leading to hypermutation can accelerate cancer development. Clinically, this subset with hypermutation may be susceptible to immune checkpoint blockade.

## INTRODUCTION

Colorectal cancer (CRC) is the third-most common cancer and the fourth-most common cause of cancer-related deaths worldwide [1]. It is generally divided into three broad categories based on hereditary influence and cancer risk: sporadic CRCs (60%), which are related neither to any family history nor to any identifiable germline mutations leading to CRC development; familial CRCs (30%), in patients that have at least one blood relative with CRC or an adenoma but still without any clear pattern of inheritance or germline mutations leading to CRC development; and hereditary CRC syndromes (10%), which result from the germline inheritance of mutations in cancer-susceptibility genes [2].

Based on age at occurrence, CRCs can also be categorized as early or late onset. Early-onset CRCs (EOCRCs) are generally defined as CRCs that occur before the age of 50 years [3]. The prevalence of EOCRCs among CRCs, typically ranges from 3% to 17% [4]. The incidence of EOCRCs has increased annually by 1.5% in men and by 1.6% in women [5]. EOCRCs occur predominantly in the distal colon (80%), particularly in the sigmoid colon and rectum, with aggressive histologic features such as signet ring cell differentiation, venous invasion, and perineural invasion [6].

EOCRCs may have a genetic predisposition or may have biological or genomic features distinct from late-onset CRCs (LOCRCs). Berg et al. performed copy number variation (CNV) and mRNA expression analyses on 23 EOCRC cases without known hereditary syndromes as well as on 17 LOCRC cases, and found that 10 genomic loci were more frequently altered in EOCRCs than in LOCRCs and that seven genes (*CLC*, *EIF4E*, *LTBP4*, *PLA2G12A*, *PPAT*, *RG9MTD2*, and *ZNF574*) were differentially expressed between EOCRCs and LOCRCs [7]. Chang et al. performed histologic, molecular, and immunophenotypic analysis on 55 sporadic EOCRC cases, and reported that a *KRAS* mutation was present in only 4% of EOCRCs; no EOCRCs possessed a *BRAF* V600E mutation [6]. Tanskanen et al. analyzed 38 EOCRC cases from a Finnish population using targeted sequencing but did not find any germline predisposition conditions other than known hereditary syndromes [8]. These studies indicate that sporadic EOCRCs may have distinct somatic, but not germline, aberration patterns.

In this study, we aimed to identify somatic aberrations that are distinct in MSS EOCRCs. One of our key motivations was that no previous study has extensively analyzed somatic aberrations in MSS EOCRCs. We therefore excluded EOCRCs with any familial history or with a mismatch repair (MMR) deficiency (i.e., microsatellite instability). *POLE* (DNA polymerase, epsilon, catalytic subunit) mutations have been reported in germline and somatic CRCs, but not in MSS EOCRCs [9].

We performed a whole exome sequencing (WES) analysis on 28 cases of MSS EOCRC (diagnosed at <40 years old to be more stringent for initial discovery) and further validated our findings in expanded cohorts of 83 MSS EOCRCs (age of onset <50 years old, the conventional criterion for EOCRCs).

## RESULTS

### The mutational pattern of hypermutated EOCRC suggests a hypermutation mechanism other than microsatellite instability

To identify novel carcinogenic mechanisms in EOCRC, we performed WES analysis on 28 tumor-normal pairs of polyposis-unrelated MSS EOCRC. The clinicopathological features of the 28 cases are summarized in Table 1. None of these cases had any familial history of CRC or microsatellite instability.

**Table 1:** Clinicopathologic features of the 28 MSS EOCRCs

Case	Sex	Age	Site	Histologic type	Differentiation	Stage	T stage	N stage	Recur	POLE
C100090	F	31	R	A	MD	IV	3	2a	yes	W
C102969	M	32	AC	A	MD	IIIB	3	1a	no	M
C081187	M	33	AC	A	MD	IIA	3	0	no	M
C092389	F	33	R	A	WD	IV	3	0	yes	W
C101770	M	33	SC	A	MD	IIB	4	0	no	W
C090634	F	34	R	A	MD	I	1	0	no	W
C091229	M	34	SC	A	MD	IIIC	3	2a	yes	W
C080637	M	35	R	A	MD	IIIC	3	2b	yes	W
C080748	F	35	C	A	MD	IIA	3	0	yes	W
C081530	M	35	R	A	MD	IIA	3	0	no	W
C102565	F	35	AC	A	WD	IIIB	3	1	no	W
C115326	M	35	R	A	MD	IIA	3	0	no	W
C115941	M	35	AC	A	MD	IIB	4	0	no	M
C102752	F	36	R	A	MD	IIIB	3	1	no	W
C061463	M	37	C	A	WD	I	2	0	no	W
C061386	M	38	SC	A	MD	IIA	3	0	no	W
C071710	M	38	AC	A	MD	IIA	3	0	no	W
C071830	M	38	C	M	MD	IIA	2	0	no	M
C090063	M	38	TC	A	MD	IIA	3	0	no	M
C101287	M	38	R	A	WD	IIA	3	0	no	W
C113929	F	38	R	A	MD	IIA	3	0	no	W
C116121	F	38	DC	A	MD	IIIB	4a	1a	yes	W
C116255	F	38	SC	A	PD	IV	4b	2b	yes	W
C050246	F	39	DC	M	MD	IIIB	3	1a	no	W
C091575	M	39	R	A	WD	I	2	0	no	W
C102887	M	39	R	A	MD	IIIC	3	2b	no	W
C113558	F	39	AC	A	MD	IV	3	2b	yes	W
C110138	F	30	C	A	MD	IIIB	3	1	no	M

Sites: Rectum = R, Ascending colon = AC, Sigmoid Colon = SC, Cecum = C, Transverse Colon = TC, Descending Colon = DC

Histologic types: Adenocarcinoma = A, Mucinous Carcinoma = M

Differentiation: WD = well-differentiated, MD = moderately differentiated, PD = poorly differentiated

*POLE*: W = Wild, M = Mutated

**Figure 1:**
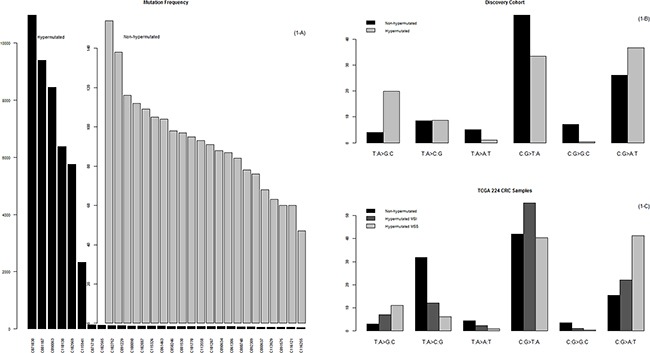
Mutational frequency and patterns **A.** The prevalence of somatic mutations in the 28 MSS EOCRCs. Here, somatic mutations are represented by nonsynonymous mutations, stop-gain mutations, stop-loss mutations, splicing variants, and indels that lead to changes in the primary structure of proteins. **B.** Mutation patterns of the 28 MSS EOCRCs. The mutation patterns were different between the hypermutated and the nonhypermutated cases. **C.** Mutation patterns of 224 CRCs in the TCGA study. Of note, the mutation patterns of the hypermutated MSI CRCs were different from those of the hypermutated MSS CRCs, which is characterized by a high C:G>A:T peak. This peak was also characteristic of the hypermutated MSS EOCRCs in our study (B).

The number of somatic mutations varied significantly among the cases in our study. In 22 of these 28 cases, the number of somatic mutations ranged from 47 to 154; however, in 6 cases, the number ranged from 2325 to 10,973, representing a rate 100 times greater, on average, than the rate found in the other 22 cases (Figure 1A). On average, nonhypermutated cases had 74 nonsynonymous mutations, 6 truncation mutations, 2 splicing variants, and 6 frame shift insertions or deletions; hypermutated cases had 6226 nonsynonymous mutations, 834 truncation mutations, 106 splicing variants, and 25 frame shift insertions or deletions (Supplementary Table S1). Since all of our EOCRC cases were MSS, we set out to further elucidate the underlying mechanism of the high mutational burden, as it was not microsatellite instability.

**Figure 2:**
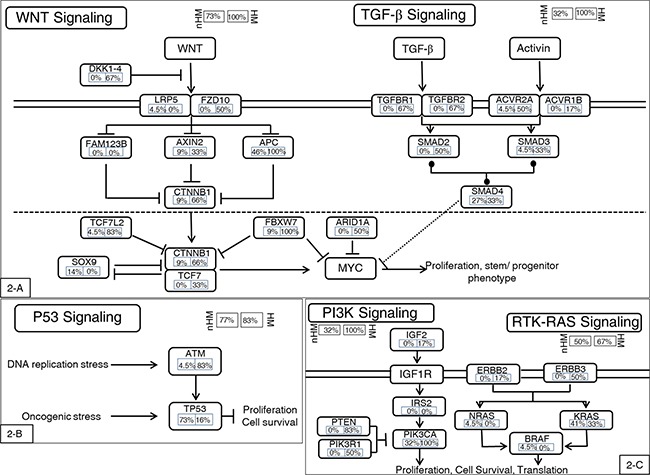
Highly mutated signaling pathways In general, the hypermutated group had higher mutational frequencies in most genes in the affected pathways. One exception is the *P53* mutation, a late-stage event in CRC carcinogenesis. The hypermutated group had low mutational frequency in P53, indicating that this group may have a carcinogenic mechanism distinct from the nonhypermutated group. **A.** WNT and TGF-β pathways. **B.** P53 pathway. **C.** PI3K and RTK-RAS pathways. Alteration frequencies are expressed as a percentage of all cases. HM: hypermutated group; nHM: nonhypermutated group.

We divided our 28 cases into two groups, hypermutated and nonhypermutated, according to their mutational burden, and compared the nucleotide changes between these two groups. As summarized in Figure 2, the C:G>A:T transversion rate was 10% more frequent and the C:G>T:A transition rate was 16% less frequent in hypermutated than in nonhypermutated cases. Then we compared the mutation patterns of our current hypermutated cases with those from the previous TCGA CRC study (hereafter referred to as the TCGA study [10]). The TCGA study included 35 hypermutated cases; 30 were microsatellite instability (MSI) CRCs, and the remaining 5 were MSS CRCs. Unlike the 30 MSI CRCs with hypermutation, the 5 MSS CRCs with hypermutation showed a high prevalence of C:G>A:T transversions (Figure 1C), which is concordant with the mutation patterns of the 6 MSS CRCs with hypermutation in our current study (Figure 1B).

### Recurrent somatic mutations in the hypermutated and nonhypermutated groups

The mutational landscape of the nonhypermutated cases in our present study was similar to that of the TCGA CRC study [10]. Frequently mutated genes included *TP53* (72.73%), *APC* (45.5%), *KRAS* (40.9%), *PIK3CA* (31.8%), and *SMAD4* (27.3%). We also found mutations in *FBXW7* (9%), *TCF7L2* (4.5%), and *NRAS* (4.5%). The most frequently mutated pathways included the *WNT* signaling pathway, the phosphoinositide 3-kinase (*PI3KCA*) signaling pathway, and the *TGF-Δ* signaling pathway (Figure 2). The mutational landscape of the hypermutated cases was more complex, due to their high mutational burden. By comparison, the hypermutated group showed a lower frequency of *TP53* mutations and a higher frequency of phosphoinositide 3-kinase (*PI3KCA*) pathway activation than the nonhypermutated group (Figure 2).

Although we started with MSS EOCRC cases, we investigated mutations in MMR genes in the hypermutated cases. Mutations in the *MSH3*, *MSH6*, *MSH2*, *Exo1*, *MLH1*, *MLH3*, *PMS1,* and *PMS2* genes lead to mismatch repair deficiency and microsatellite instability [11]. Hence, we first investigated the mutational status of these eight genes in our six hypermutated cases. In total, we identified 30 mutations in these eight genes. However, when we searched for these mutations against the MMR gene mutation databases (e.g., Leiden Open Variation Database [LOVD] - human mismatch repair genes [12]; MMR Gene Unclassified Variants Database [13]), only S44F in the *MLH1* gene had been previously reported, indicating that most were passenger mutations caused by an accelerated mutational process (Supplementary Table S2). In summary, we found no convincing evidence of an MMR deficiency in our six hypermutated cases. Based on this finding, we set out to further elucidate the mechanisms underlying the high mutational burden, beyond MSI or MMR deficiency.

### The *POLE* mutation is associated with hypermutated EOCRCs

We found that the *POLE* gene was mutated in all six hypermutated cases in our study but not in the nonhypermutated cases (6/28, 21%). Among our six hypermutated cases, four had the same Proline(P) 286 Arginine(R) mutation, located in the exonuclease domain of the *POLE* gene; the other two cases had either I1925T or R1382C mutations, located outside the exonuclease domain of the *POLE* gene. Mutations in the exonuclease domain of the *POLE* gene, including P286R, cause hypermutation [14]. However, the reported frequency of the *POLE* P286R mutation in CRC is extremely low. For example, in the TCGA CRC study, no *POLE* P286R mutation was reported in any of the 224 CRC cases [10]. Notably, most CRC cases analyzed in the TCGA study were LOCRCs (excluding 15 cases). In our present analysis of the MSS EOCRCs, the frequency of *POLE* P286R was 14% (4/28). Based on this finding, we hypothesized that the early acquisition of *POLE* P286R may lead to somatic hypermutation, which in turn accelerates cancer development. This hypothesis may partly explain why the frequency of *POLE* P286R mutation is extremely high in MSS EOCRCs.

To validate this hypothesis, we screened for the presence of a *POLE* P286R mutation in the 83 MSS EOCRCs (age of onset <50 years) and 27 MSS LOCRCs, using Sanger sequencing. We found that 7.2% of EOCRCs (6/83) had a *POLE* P286R mutation; in contrast, no LOCRCs had a *POLE* P286R mutation.

Using targeted capture sequencing of 504 cancer-related genes, we further validated whether these six MSS EOCRCs with a *POLE* P286R mutation were hypermutated. These six MSS EOCRCs had, on average, ∼16 times more mutations than the average number of mutations in other cancers. The scale of the difference in the number of mutations (∼16-fold) observed in the targeted capture sequencing is compatible with the ∼100-fold difference observed in the aforementioned WES analysis, given the difference of genome coverage between the two different analytical platforms. The mutation patterns for these six samples featured a high C:G>A:T transversion rate, like those of the initial six hypermutated samples.

### Clinicopathological features of *POLE*-mutated EOCRCs

**Figure 3:**
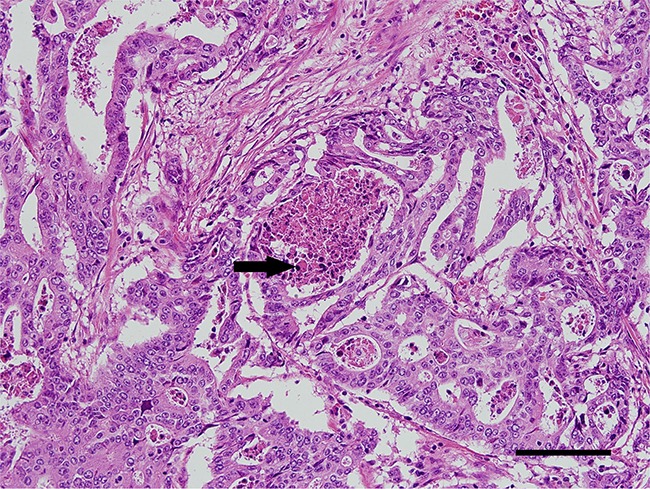
A representative immunohistochemistry image of POLE-mutated colorectal cancer Tumor cells form cribriform architectures that contain apoptotic debris in their lumen (arrow). (Hematoxylin and eosin, ×20 objective lens, scale bar = 120 μm).

**Figure 4:**
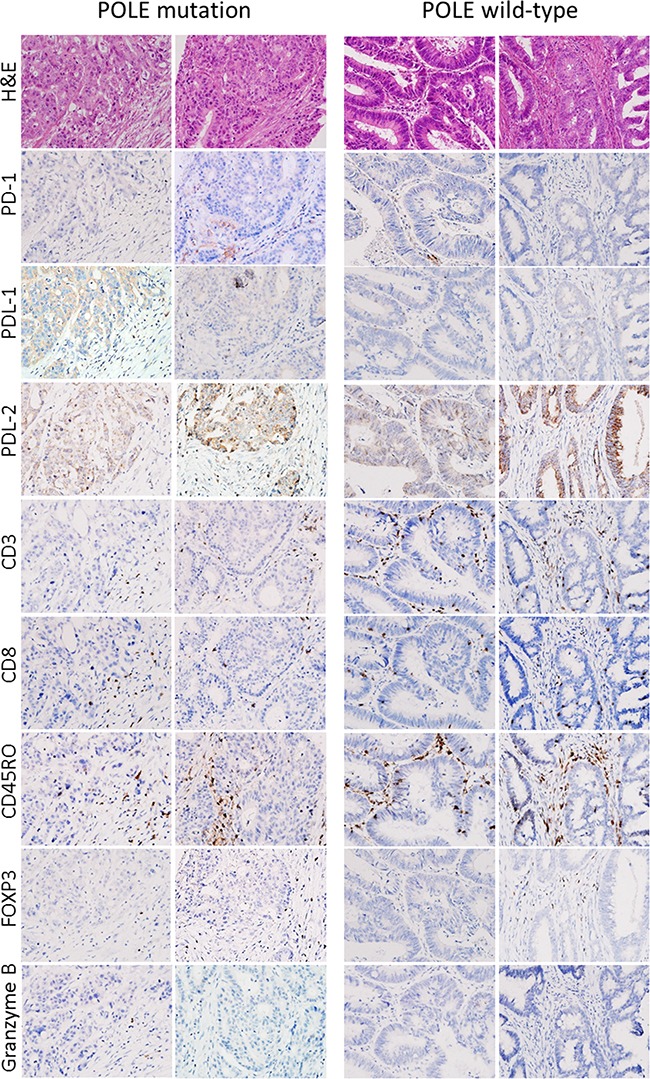
Representative immunohistochemical results Immunohistochemical analyses of immune-related marker expression in tumor cells and tumor-infiltrating lymphocytes (TILs) using five cytotoxic T cell markers and immune checkpoint molecules. TILs were mostly positive for CD45ro and/or CD3, and some TILs were positive for PD-1.

As previously described, EOCRCs have distinct clinicopathological features [6]. Because we identified a new subset of EOCRCs with hypermutation, we investigated whether this new subset has clinicopathological features distinct from the other EOCRCs without hypermutation. All CRCs harboring a *POLE* mutation were from patients younger than 50 years old at the time of diagnosis. CRCs with a *POLE* mutation also occurred far more frequently on the right side of the large intestine than on the left side and affected men more frequently than women (Table 2). Microscopically, *POLE*-mutated tumors did not show any peculiar histologic subtype, such as mucinous or signet ring cell carcinoma, but they frequently formed cribriform structures and intraluminal necrotic debris, which contained neutrophils and apoptotic bodies (Figure 3). Intratumoral or peritumoral inflammatory cell infiltration was not prominent. Since *POLE*-mutant endometrial carcinomas have been reported to have increased immunogenicity and to elicit intratumoral cytotoxic T cell infiltration [15–17], we performed immunohistochemical analysis of immune-related marker expression in tumor cells and tumor-infiltrating lymphocytes (TILs) using five cytotoxic T cell markers and immune checkpoint molecules. Unexpectedly, the numbers of TILs showing expression of cytotoxic T cell-related markers were not significantly different between *POLE*-mutant and *POLE*-wild-type CRCs (Figure 4). TILs were mostly positive for CD45ro and/or CD3, and some TILs were positive for PD-1. *PD-1*-positive immune cells tended to be greater in *POLE*-mutant CRCs than in *POLE*-wild-type CRCs (Supplementary Figure S1, p = 0.157 [Supplementary Table S5]). Likewise, *PD-L1* expression in tumor cells was not significantly different between *POLE*-wild-type and *POLE*-mutant tumors. Interestingly, the mutated POLE subset tended to show better recurrence-free survival (Supplementary Figure S2). There was no tumor recurrence after curative resection among 6 POLE-mutated EOCRC patients, in contrast with 8 out of 22 POLE-wild-type EOCRC patients (Table 1). In summary, the clinicopathological features of this new subset of EOCRCs with hypermutation were different from the common features observed in EOCRCs in general.

**Table 2:** Comparison of clinicopathologic features between MSS EOCRCs with and without a ***POLE*** mutation

Clinicopathologic variables	*POLE* mutation		*P* value*
	Absent (N=84)	Present (N=11)	
**Age (years)**			0.900
<40	70 (83.3%)	9 (81.8%)	
≥40	14 (16.7%)	2 (18.2%)	
**Sex**			0.042
Male	50 (59.5%)	10 (90.9%)	
Female	34 (40.5%)	1 (9.1%)	
**Site**			<0.001
Right side	12 (14.3%)	8 (72.7%)	
Left side	72 (85.7%)	3 (27.3%)	
**Pathologic diagnosis**			0.914
Adenocarcinoma	70 (83.3%)	10 (90.9%)	
Mucinous carcinoma	12 (14.3%)	1 (9.1%)	
Signet ring cell carcinoma	1 (1.2%)	0 (0%)	
Adenosquamous carcinoma	1 (1.2%)	0 (0%)	

* *P* value by two-sided χ2 test.

## DISCUSSION

We investigated the distinct carcinogenic mechanisms that accelerate cancer development in MSS EOCRCs. Our main finding was that MSS EOCRCs are a distinct subclass of CRC, with hypermutation associated with a *POLE* P286R mutation. Our findings indicate that a somatic *POLE* P286R mutation is a frequent carcinogenic driver in EOCRCs, leading to the rapid accumulation of additional somatic mutations, thus accelerating cancer development.

The *POLE* gene encodes the catalytic subunit of the DNA polymerase epsilon, which is involved in DNA repair and chromosomal DNA replication [18] and has three PFAM domains: the exonuclease domain, DNA polymerase family B, and DUF1744 (PFAM). According to recent studies, somatic and germline mutations in the exonuclease domain of the *POLE* gene are important carcinogenic drivers. For example, the germline mutation in *POLE* L424V induces a predisposition to CRC [19, 20]. Somatic or germline mutations in the exonuclease domain of the *POLE* gene cause hypermutated CRCs (∼3%) or endometrial cancers (∼7%) [14, 21]. Yeast and mice with mutations in the exonuclease domain of *POLE*, or its homologues, show an increase in the rate of spontaneous mutations [22]. *POLE* P286R, an exonuclease domain mutation, has been functionally validated in a yeast model and has shown a strong mutator phenotype, comparable with complete MMR deficiency [21].

The chances of new mutations during genome replication are limited by replicase accuracy in base selection and nearly instant proofreading of replication errors and post-replicative MMR [23]. The deactivation of any of these safeguards can lead to hypermutation. Similarly, mutations can also be introduced during replication when error-prone TLS polymerases copy small stretches of DNA that do not contain lesions [24]. Main three reasons of hypermutation are as follow:

1. MMR defects: Germline genetic defects in one of the MMR genes (*MSH2*, *MSH6*, *PMS2*, or *MLH1*) can lead to hereditary predisposition to nonpolyposis CRC as well as to several other types of cancers. MMR defects usually cause microsatellite instability, which can easily be detected in WGS or WES datasets [25].
2. *POLE* and DNA polymerase delta (*POLD*) mutations: Somatic mutations in the respective catalytic subunits of replicative DNA polymerases epsilon (*POLE1*) and delta (*POLD1*) have recently been associated with familial predisposition to colorectal, endometrial, and ovarian endometroid cancers [14]. *POLE* has intrinsic proofreading activity. Current evidence indicates that a defect in this activity due to mutation leads to a loss in replication fidelity, substantially increasing the mutational burden. *POLE* mutations specifically in EDM affect conserved residues, resulting in impaired intrinsic proofreading activity, loss of replication fidelity, and a substantial increase in the mutational burden, accumulating a substantial number of driver mutations in less than 6 months [26].
3. TLS polymerases: TLS DNA polymerase eta can perform error-free copying of cyclobutane pyrimidine dimers, but it has a high error rate when copying an undamaged template *in vitro* [27].

In the conventional adenoma-carcinoma model of CRC genesis, *TP53* mutation occurs in the late stage of carcinogenesis when late adenomas develop into carcinomas [28]. The relatively low frequency of *TP53* mutations in the hypermutated group may be additional supporting evidence that EOCRCs with the *POLE* mutation have a distinct carcinogenic mechanism. It has been reported that the expression of a dominant active PI3K synergizes with the loss of *APC* activity, resulting in dramatic changes in tumor multiplicity, size, morphology, and invasiveness [29]. In other words, the dominant active PI3K is able to initiate the development of adenocarcinomas in the colon via a noncanonical mechanism of tumorigenesis [30]. In summary, hypermutated CRCs are caused via a noncanonical mechanism of tumor initiation that is mediated through activation of PI3K and not through aberrations in WNT signaling.

Despite the functional importance of *POLE* mutations, the observed frequency of somatic *POLE* mutations in CRC has been extremely low. In the TCGA CRC study, a *POLE* mutation was reported in only 15 of 224 cases (approximately 7%). Among these cases, seven (∼3%) had mutations in the exonuclease domain (Supplementary Table S3). All samples with an exonuclease domain mutation were hypermutated. Although no *POLE* P286R mutations were reported in the TCGA study, there was one mutation (P286H) at the same amino acid residue [10]. In another CRC exome sequencing study, 2 of 74 (3%) cases had *POLE* P286R mutations [31]. Kane and Shcherbakova [21] also reported that 1 of 52 CRC cases (∼2%) displayed an ultramutator phenotype with a *POLE* P286R mutation. In our current study, the frequency of the *POLE* P286R mutation was ∼9% in MSS EOCRCs, which is at least three times greater than any observed frequency in CRCs.

Our current findings provide a clinically meaningful link between MSS CRCs and a recent therapeutic breakthrough in cancer immunotherapy. Recently, Le et al. [32] reported that CRCs with high mutational burden due to MMR deficiency are susceptible to immune-checkpoint blockade. In our current analyses, we identified a subset of MSS CRC that is hypermutated due to mutations in the *POLE* gene; however, we did not find any striking differences in the immune profiles of tumor cells and TILs between *POLE*-mutant and *POLE*-wild-type CRCs, unlike cases with an MMR deficiency. This lack of a clear profile difference may be attributable either to an inadequate number of cases analyzed in our present report or to the different mutator phenotype of a *POLE* mutation compared to that of MMR deficiency. Thus, it remains to be investigated further whether this subset of MSS CRCs with hypermutation is also susceptible to immune checkpoint blockade. However, it may be at least possible to efficiently identify CRC patients with *POLE* mutations by selecting affected patients with an extremely early onset age. For further clarification we also checked the insilico immunogenicity against MHC class 1 of our mutated proteins using the IEDB Analysis Resource [33]. Approximately 50% of all peptides were immunogenic (Supplementary Table S7, Supplementary Figure S3); a possible reason for similar immune profiles in our experiments was the rapid accumulation of a high number of somatic mutations, in less than 6 months [26].

In summary, we focused on the *POLE* P286R mutation for three reasons. First, *POLE* P286R is one of only a few *POLE* exonuclease domain mutations to be functionally validated to cause hypermutation [22]. Second, the *POLE* P286R mutation was the only mutation found in the exonuclease domain in our study. Third, even in endometrial cancers in which the mutational frequency of *POLE* is relatively high, the mutational frequencies of other mutations in the exonuclease domain of *POLE* are extremely low (i.e., usually <5%) (Supplementary Table S4) [34]. This does not exclude the importance of other *POLE* mutations in EOCRCs, which will be addressed in our future research.

## MATERIALS AND METHODS

### Patients and tumor specimens

For the initial discovery analysis, using WES and a CNV chip, we used matched normal-tumor samples from 28 Korean patients with colorectal adenocarcinoma without either a clinical history or gross pathologic features of polyposis. The 28 colorectal adenocarcinomas presented no evidence of microsatellite instability in the Bethesda panel. For validation, we used specimens from an additional 83 EOCRC and 27 LOCRC patients. All patients had completely annotated clinical data, including overall survival, recurrence, histologic subtype, clinical stage, tumor stage, and microsatellite status. The specimens and data used in this study were obtained from the Asan Bio-Resource Center of the Korea Biobank Network with the approval of the Institutional Review Board of the Asan Medical Center (Seoul, South Korea).

### WES of 28 matched normal-tumor samples

For the tumor samples, a frozen section from each sample was subjected to hematoxylin and eosin (H&E) staining and histologic examination to determine tumor coverage and cellularity. DNA was extracted for further analysis when tumor coverage was ≥80% and tumor cellularity was ≥50%. Genomic DNA was extracted using the QIAamp DNA formalin-fixed paraffin-embedded (FFPE) Tissue Kit (Qiagen Inc., Valencia, CA), according to the manufacturer's instructions. After elution in DNase-/RNase-free water, genomic DNA was quantified using the NanoDrop spectrophotometer and PicoGreen system (Invitrogen Life Technologies, Carlsbad, CA). Whole exon capture and sequencing library preparation were performed using the SureSelect All Exon 50 Mbp Kit (Agilent, Santa Clara, CA), according to the manufacturer's instructions. The quality of the amplified fragment libraries was verified by capillary electrophoresis, using the Agilent Bioanalyzer (Agilent). Cluster generation in the flow cells was achieved using the cBot automated cluster generation system (Illumina, San Diego, CA). Then paired-end DNA sequences 100 bp in length were obtained with the Illumina HiSeq 2000 platform (Illumina).

### Targeted sequencing

An additional 110 CRC cases were analyzed to screen for the P286R mutation in exon 9 of *POLE* (NM.006231). After peer review (by a pathologist) of matched H&E slides for each of the FFPE tissues under the microscope, two to five 6 μm sections were used for extraction of genomic DNA per FFPE tissue specimen, depending on tumor size and cellularity. After treatment with xylene and ethanol for de-paraffinization, genomic DNA was isolated using the NEXprep FFPE Tissue Kit (#NexK-9000; Geneslabs, Gyeonggi, Korea) in accordance with the manufacturer's recommendations. The tissue pellet was completely lysed overnight at 56°C by incubation with proteinase K in lysis buffer, followed by an additional incubation for 3 min with magnetic beads and solution A at room temperature. After incubation for 5 min on the magnetic stand, the supernatant was removed, and then washed with ethanol three times. After the beads were dried for 5 min, DNA was eluted in 50 μL DNase-/RNase-free water, followed by quantification using the Quant-iT™ PicoGreen dsDNA Assay kit (Invitrogen Life Technologies). Exon 9 was amplified with the following primers: 5′-CTCCCTGTTGGTGATGAGGT-3′ (forward) and 5′-GGGTCCTTCTCCCAGCTCTA-3′ (reverse). The Sanger sequencing of all PCR products was subsequently conducted on an ABI Prism 3730xl Genetic Analyzer (Life Technologies, Carlsbad, CA).

For additional validation of P286R mutation and overall mutation burden, targeted next-generation sequencing was performed using the MiSeq platform (Illumina), with OncoPanel version 2 (OP_v2) (Agilent, custom-ordered), to capture the exons of 505 cancer-related genes plus partial introns from 15 genes often rearranged in cancer. Samples (200 ng) of gDNA were fragmented by sonication (Covaris Inc., Woburn, MA) to an average size of 250 bp, followed by size selection using Agencourt AMPure XP beads (Beckman Coulter, Inc., Fullerton, CA). A DNA library was prepared by ligation of 50 ng purified DNA with the TruSeq adaptor using a SureSelect XT Reagent Kit (Agilent Technologies, Glostrup, Denmark). Each library was constructed with 6 bp sample-specific barcodes, quantified using PicoGreen, and four libraries were pooled to a total of 600 ng for hybrid capture using the Agilent SureSelectXT custom kit (OP_v2 RNA bait, 2.9 Mb; Agilent Technologies). The concentration of the enriched target was measured by quantitative PCR (Kapa Biosystems, Inc., Wilmington, MA) and loaded onto an MiSeq (Illumina) for paired-end sequencing.

### MSI testing

The MSI status of tumor samples was secured from clinical records. As a clinical MSI test, DNA from normal and tumor tissue was PCR amplified using a primer set for the five-marker Bethesda panel (BAT25, BAT26, D5S346, D2S123, and D17S250), and products were run on an ABI Prism 310 DNA sequencer (Perkin-Elmer Applied Biosystems Division, Foster City, CA) and analyzed using GeneScan version 3.1 software (Perkin-Elmer Applied Biosystems Division). Tumors were classified as MSI-H, two or more unstable markers; MSS, no unstable markers; or low-frequency MSI (MSI-L).

### Bioinformatics analysis

Sequenced reads were aligned to the human reference genome (NCBI build 37) using BWA (0.5.9) [35] with default options. To remove PCR duplicates from the aligned reads, we used MarkDuplicates from the Picard package. De-duplicated reads were realigned at known indel positions with the GATK IndelRealigner [36]. Then base qualities were recalibrated using the GATK TableRecalibration. Somatic single-nucleotide variants and short indels were detected with unmatched normals using Mutect [37] and SomaticIndelocator in GATK [36]. Common and germline variants from the candidates of somatic variants were filtered out with common dbSNP (141 found in ≥1% of samples) and a panel of normals.

### Tissue microarray construction and immunohistochemical analysis

For immunohistochemical analyses, a tissue microarray (TMA) was constructed, using FFPE tissue blocks from the 28 resected EOCRC tissues. TMA sections were immunostained using an automated staining device (Benchmark XT; Ventana Medical Systems, Oro Valley, AZ). Briefly, whole tissue sections (4 μm thick) were transferred to poly-L-lysine-coated adhesive slides and dried at 74°C for 30 min. After epitope retrieval by heating for 1 h in ethylenediaminetetraacetic acid (pH 8.0) in the autostainer, the samples were incubated with primary antibodies: anti-CD3 (1:600, DAKO, Carpinteria, CA), anti-CD4 (1:4, Ventana Medical Systems), anti-CD20 (1:800, DAKO), anti-CD8 (1:400, DAKO), anti-FOXP3 (1:100, Abcam, Cambridge, MA), anti-Granzyme B (1:50, Cell Marque Corp., Rocklin, CA), anti-PD1 (1:1000, Cell Marque Corp.), and anti-PD-L1 (1:25, Cell Signaling Technology Inc., Danvers, MA). The sections were subsequently incubated with the appropriate secondary antibodies and then visualized using the UltraView Universal DAB Detection kit (Ventana Medical Systems). Nuclei were counterstained with Harris hematoxylin.

Immunostained TMA slides were scanned using a Vectra digital slide scanner (PerkinElmer, Waltham, MA) and quantitative analysis for immune-related marker expression was performed using inForm image analysis software (PerkinElmer), according to the manufacturer's instructions.

## SUPPLEMENTARY MATERIALS FIGURES AND TABLES
















