# Supplementary material for: The somatic POLE P286R mutation defines a unique subclass of colorectal cancer featuring hypermutation, representing a potential genomic biomarker for immunotherapy
Source: Oncotarget. 2016 Sep 6;7(42):68638–49. doi: 10.18632/oncotarget.11862 (PMC5356579; doi:10.18632/oncotarget.11862)
Supplement: Supplementary file 1 [file oncotarget-07-68638-s001.pdf]

The somatic *POLE* P286R mutation defines a unique subclass of colorectal cancer featuring hypermutation, representing a potential genomic biomarker for immunotherapy

SUPPLEMENTARY FIGURES AND TABLES

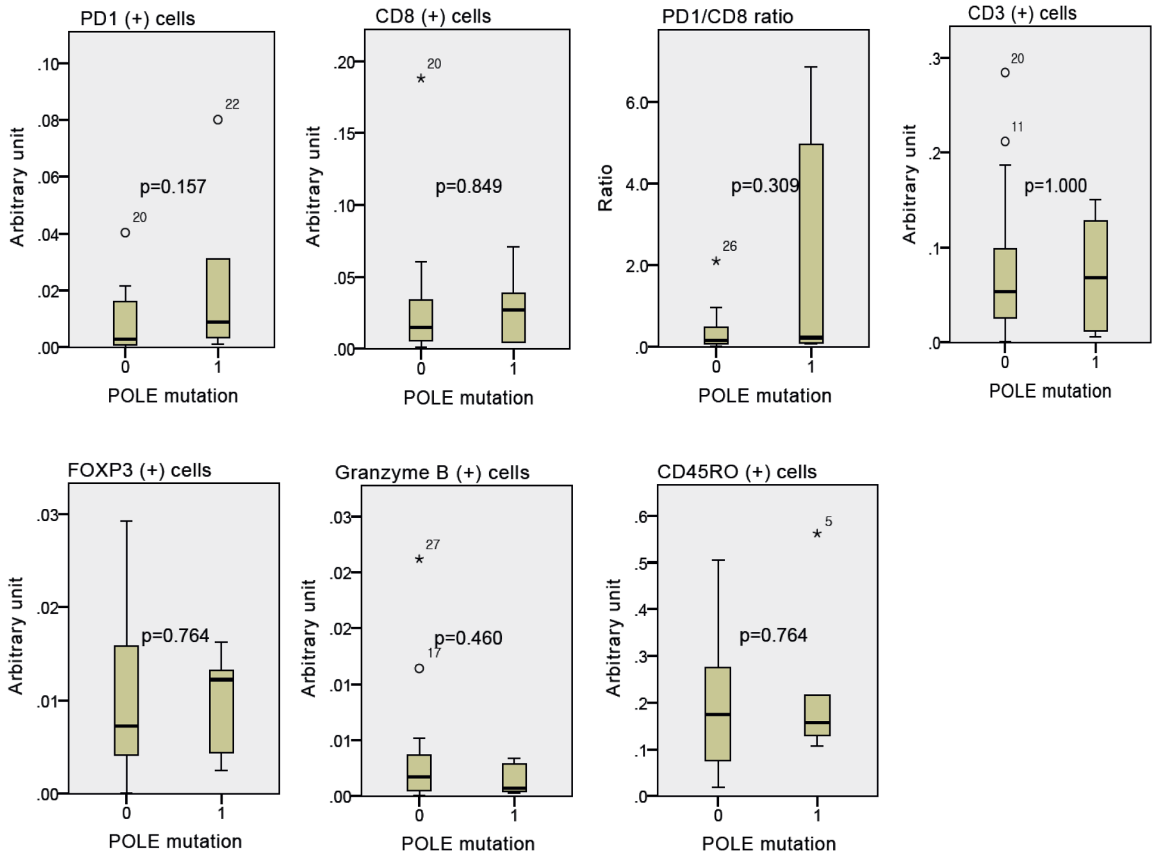

Supplementary Figure S1: Quantitative measurements of tumor-infiltrating lymphocyte subsets. No statistically significant differences were found but the number of PD1-positive cells was slightly higher in POLE-mutant tumors (labeled "1") than in POLE-wild-type tumors (labeled "0"). All counts along the Y-axis were normalized values against the total number of counted nuclei. (Horizontal line in the middle of each box: median; boxes: 25th percentile to 75th percentile; whiskers: 1.5 × interquartile range from the boundary of each of the boxes; circles: outlier values with corresponding case numbers; p-value by Mann-Whitney U test, 2-tailed.)

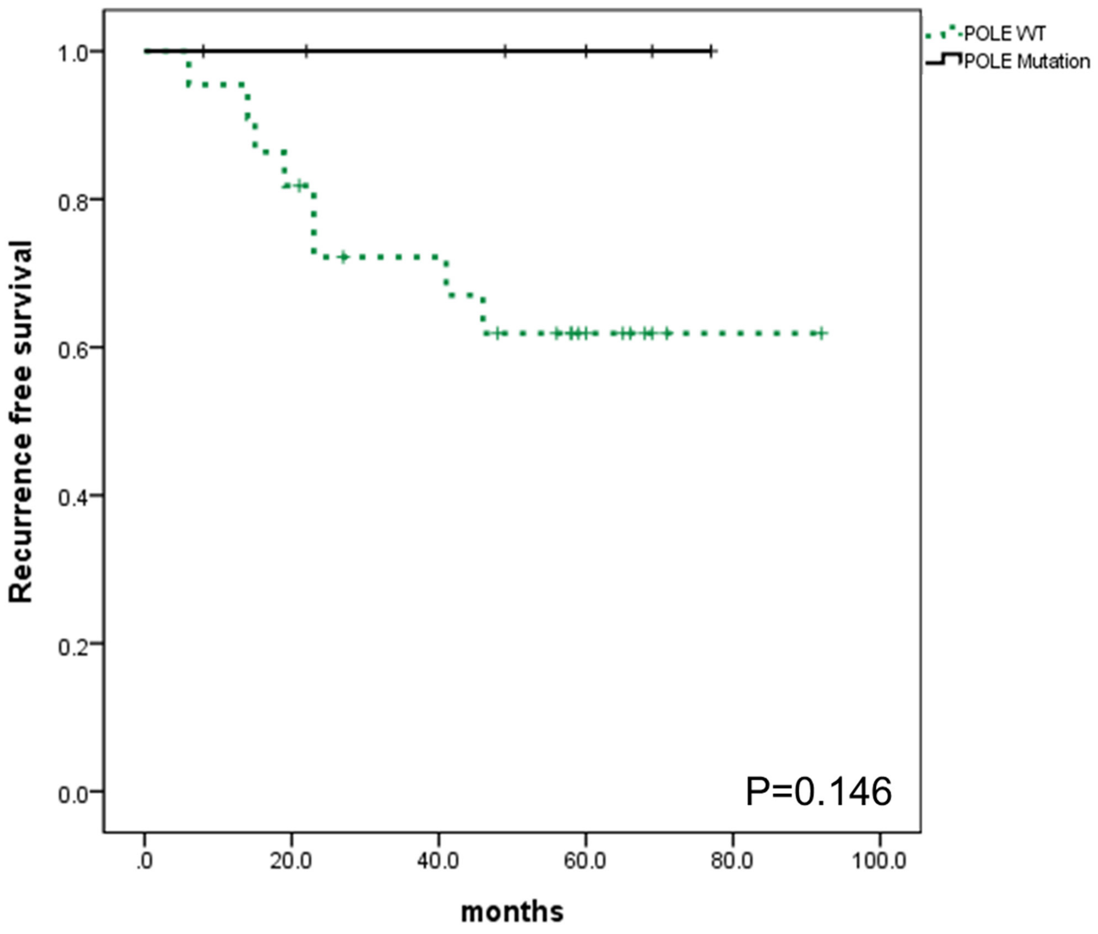

Supplementary Figure S2: Recurrence-free survival for *POLE*, mutant, and wild type.

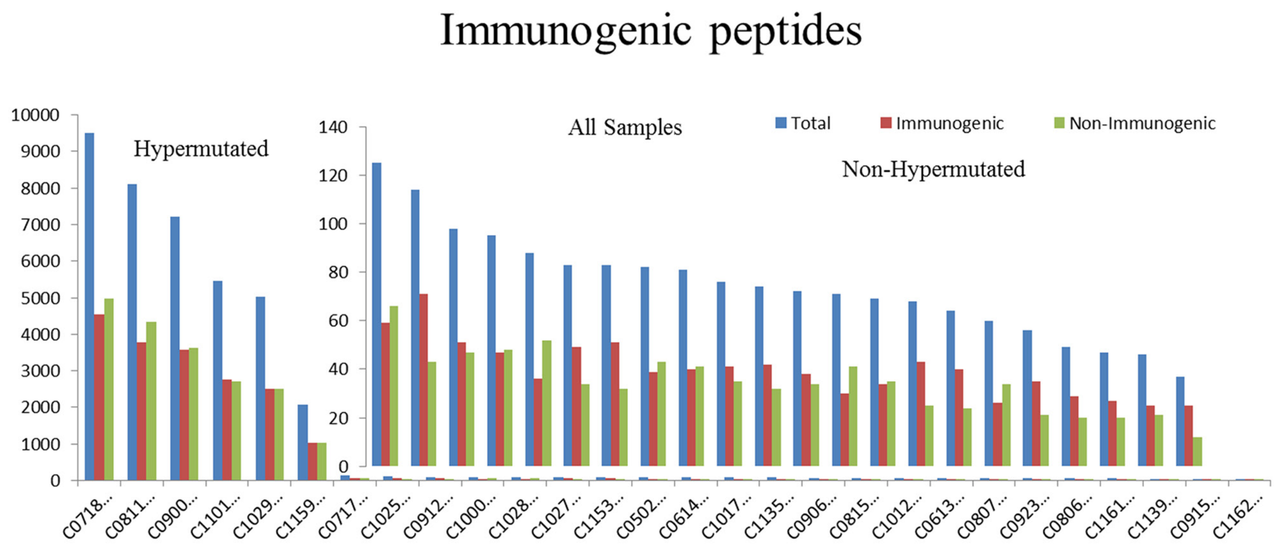

Supplementary Figure S3: Insilico estimation of immunoscores of all nonsynonymous mutations.

Supplementary Table S1: Mutation summary of hypermutated and nonhypermutated samples. This includes the number of nonsynonymous, stopgain, frameshift, and splicing mutations. It also includes the number of mutations present in ClinVar, dbSNP, and COSMIC.

See Supplementary File 1

Supplementary Table S2: The number of mutations found in MMR genes with predicted effects and status in LOVD and MMR-DB.

See Supplementary File 2

Supplementary Table S3: Mutations of the *POLE* gene in the TCGA CRC study.

See Supplementarty File 3

Supplementary Table S4: Mutations of the *POLE* gene in the TCGA endometrial cancer study.

See Supplementarty File 4

Supplementary Table S5: The distribution of PD-L1 immunoscores depending on POLE mutation status.

See Supplementarty File 5

Supplementary Table S6: Mutation master table for all 28 samples.

See Supplementarty File 6

Supplementary Table S7: Immunoscores of all nonsynonymous mutations.

See Supplementarty File 7
